# Synchronization of Fractional-Order Complex Chaotic Systems Based on Observers

**DOI:** 10.3390/e21050481

**Published:** 2019-05-10

**Authors:** Zhonghui Li, Tongshui Xia, Cuimei Jiang

**Affiliations:** 1 Business School, Shandong Normal University, Jinan 250014, China; 2 School of Mathematics and Statistics, Qilu University of Technology (Shandong Academy of Sciences), Jinan 250353, China

**Keywords:** complex modified projective synchronization, fractional-order complex chaotic system, nonlinear state observer, 34C28, 34D06

## Abstract

By designing a state observer, a new type of synchronization named complex modified projective synchronization is investigated in a class of nonlinear fractional-order complex chaotic systems. Combining stability results of the fractional-order systems and the pole placement method, this paper proves the stability of fractional-order error systems and realizes complex modified projective synchronization. This method is so effective that it can be applied in engineering. Additionally, the proposed synchronization strategy is suitable for all fractional-order chaotic systems, including fractional-order hyper-chaotic systems. Finally, two numerical examples are studied to show the correctness of this new synchronization strategy.

## 1. Introduction

The fractional-order complex chaotic systems (FOCCS), as a special kind of nonlinear systems, combine advantages of fractional-order real systems and integer-order complex chaotic systems, and thus have more complex and richer behavior. Furthermore, a broader application of FOCCS has been developed in cryptography and signal processing. Therefore, many scholars have devoted a lot of effort to study FOCCS and have obtained lots of useful results on the dynamic behavior, stabilization, control, and synchronization of FOCCS in recent years. As shown in [1], Gao and Yu employed numerical simulation to study chaotic characteristics of a fractional-order complex duffing oscillator. The chaotic behavior of fractional-order logistic equations with complex variables was discussed in detail in [2]. Subsequently, a large number of FOCCS, including the fractional-order complex Lorenz system [3], complex Chen system [4], complex T system [5], complex Lü system [6], and the fractional-order hyper-chaotic complex Lü system [7], have been found one after another. In the meantime, lots of meritorious results on chaos synchronization of FOCCS have been reported, and various regimes of synchronization have been presented, such as complete synchronization (CS) [3,8,9], anti-synchronization (AS) [6,10], hybrid projective synchronization [11,12], combination synchronization [13,14], combination–combination synchronization [15], etc. For other recent works on this subject, please refer to the previous literature [16,17,18,19,20,21,22,23,24,25].

Complex modified projective synchronization (CMPS) is a new type of complex synchronization based on complex chaotic systems that was proposed almost simultaneously in 2013 by Mahmoud et al. [26] and Zhang et al. [27]. CMPS means that state variables of the master system converge to state variables of the slave system with a complex constant scaling matrix. Therefore, CMPS can contain several types of synchronization, such as complex projective synchronization (CPS) [28], complex complete synchronization (CCS) [29], complex anti-synchronization (CAS) [30], modified projective synchronization (MPS) [31], projective synchronization (PS) [32], etc. In CMPS, the complex scaling factors are arbitrary and unpredictable, and the plural arithmetic is complicated, so that it is more difficult for an interceptor to extract signal information from transmitted information. What is more, as complex scaling factors build a bridge between real chaos and complex chaos, CMPS can increase the scope of synchronization, and then it can also greatly enhance the security and diversity of communications. Consequently, CMPS can have wide application in many fields, and thus it is very valuable and meaningful to study CMPS.

However, in the existing literature, most of works discuss the CMPS of integer-order complex chaotic systems [26,27,33], and there are few results concerning the CMPS of FOCCS. For instance, Jiang et al. studied the CMPS of FOCCS with incommensurate orders by employing active control [16]; Liu used the feedback control to investigate CMPS between a fractional-order complex chaotic system and a real hyper-chaotic system [17]; Tian and Zhong realized CMPS of two uncertain FOCCS by means of adaptive control strategy [18]. Furthermore, there is seldom information available about observer-based CMPS of FOCCS.

Motivated by the above discussions, this paper develops an observer-based approach to realize CMPS of FOCCS. The technique of nonlinear observer will be employed, which has been used in the study of other types of synchronization phenomena [34,35,36,37]. Compared with the previous works, the synchronization scheme and the master–slave systems studied in this paper are more general. In our synchronization scheme, if the fractional-order master system is provided, then the fractional-order slave system could be determined in the form of a state observer, and the drive signal should be chosen so that the master system satisfies certain conditions to ensure CMPS. The proposed observer-based control enables CMPS in a general class of fractional-order complex chaotic systems without the limitation of partial-linearity and does not require the computation of the conditional Lyapunov exponents. Therefore, the proposed scheme will find a wide variety of industrial applications.

The structure of this article is as below. Section 2 presents the relevant definition and lemma. In Section 3, a nonlinear state observer is constructed to realize CMPS of FOCCS. Following this method, we obtain the fractional-order error complex system, where a gain matrix can be determined by the pole placement technique to asymptotically stabilize the error system. Section 4 applies this new synchronization scheme to achieve CMPS in two examples. Finally, relevant conclusions are provided.

## 2. Problem Statement

Fractional calculus is the extension of integration and differentiation to arbitrary non-integer orders. Some typical definitions of fractional derivatives can be referred to in [38]. Due to wide engineering applications of the Caputo definition, we adopt this definition in this article. Next, we introduce the Caputo derivative.

**Definition** **1.** *[38] Given the function g(t), the Caputo fractional derivative of fractional-order q is defined as follows:*

$$\begin{array}{c} D_{*}^{q}g\left(t\right)=\frac{1}{\Gamma (n-q)}\int_{t_{0}}^{t}\frac{g^{\left(n\right)}\left(\tau \right)}{{\left(t-\tau \right)}^{q-n+1}}d\tau ,\;\;t>t_{0}, \end{array}$$

where q∈(m−1,m), m=[q]+1, [q] is the integer part of q, Γ represents the Gamma function, and $D_{*}^{q}$ indicates the q-order Caputo differential operator.

In this paper, we always suppose that *q* is a positive number less than 1 since the fractional-order *q* often lies in (0,1) in engineering. For the sake of our synchronization result, we introduced the following stability results for linear fractional differential equations. Given the autonomous system

(1) $$\begin{array}{c} D_{*}^{q}y=By, \end{array}$$

where the state variable $y\in R^{n}$ and the initial condition $y\left(0\right)=y_{0}$, system (1) has the following results.

**Lemma** **1.** *[39] System (1) is*

*(i) asymptotically stable iff*

$$\begin{array}{c} |arg(\lambda_{l}\left(B\right))|>q\pi /2,\;for\;l=1,2,3\ldots , \end{array}$$

*where $arg(\lambda_{l}\left(B\right))$ stands for the argument of the eigenvalue $\lambda_{l}$ of B. For this case, the component of the state decay converges to 0 as $t^{-q}$.*

*(ii) stable iff*

$$\begin{array}{c} |arg(\lambda_{l}\left(B\right))|\geq q\pi /2,\;for\;l=1,2,3\ldots , \end{array}$$

*and those critical eigenvalues with $|arg(\lambda_{l}\left(B\right))|=q\pi /2$ have geometric multiplicity one.*

## 3. Problem Description and Synchronization Scheme

FOCCS are generally described by a set of nonlinear differential equations. Generally speaking, a FOCCS can be divided into two major parts: one is linear and the other is nonlinear. Therefore, we study the following FOCCS:

(2) $$\begin{array}{c} D_{*}^{q}z=\Theta z+\Psi f\left(z\right)+\Omega , \end{array}$$

where the state vector $z={\left(z_{1},z_{2},\ldots ,z_{n}\right)}^{T}=z^{r}+jz^{i}\in C^{n}$, $z^{r}$ is the real part of *z* and $z^{i}$ is the imaginary part of *z*. $\Theta \in R^{n\times n}$ and $\Psi \in R^{n\times m}$ are real matrices, $\Omega \in R^{n\times 1}$(or $\Omega \in C^{n\times 1})$, and $f={\left(f_{1},f_{2},\ldots ,f_{m}\right)}^{T}$ ($f_{i}$ stands for complex nonlinear function) are column vectors.

**Remark** **1.** *System* (2) *can describe lots of FOCCS, including fractional-order complex Lorenz system [3], complex Chen system [4], complex T system [5], complex Lü system [6], hyper-chaotic complex Lü system [7], etc.*

In order to realize CMPS, we take system (2) as the master system. Thus, suppose that system (2) has the following output

(3) $$\begin{array}{c} y=f(z)+Kz, \end{array}$$

where $K\in R^{n\times m}$ is a gain matrix. Given an invertible constant matrix $\Phi =\Phi^{r}+j\Phi^{i}\in C^{n\times n}$, its inverse matrix can be expressed as $\Phi^{-1}$. Thus, design the observer of FOCCS (2) as the slave system

(4) $$\begin{array}{c} D_{*}^{q}\hat{z}=\Phi^{-1}\Theta \Phi \hat{z}+\Phi^{-1}\Psi f\left(\hat{z}\right)+\Phi^{-1}\Omega +\Phi^{-1}\Psi \left(y-\hat{y}\right), \end{array}$$

and define the output in the following form:

(5) $$\begin{array}{c} \hat{y}=f\left(\hat{z}\right)+K\Phi \hat{z}. \end{array}$$

In the sequel, define the synchronization error function as

(6) $$\begin{array}{c} e\left(t\right)=z\left(t\right)-\Phi \hat{z}\left(t\right), \end{array}$$

where Φ is called a complex scaling matrix.

Thus, based on systems (2) and (4), the definition of CMPS can be stated as the following.

**Definition** **2.** *CMPS between systems* (2) *and* (4) *can be realized if there is a complex matrix $\Phi =\Phi^{r}+j\Phi^{i}=\mathrm{diag}\left\{\varphi_{1},\varphi_{2},\ldots ,\varphi_{n}\right\}\in C^{n\times n}$ satisfying*

$$\begin{array}{c} \lim_{t\to \infty }{\left||e\left(t\right)|\right|}^{2}=\lim_{t\to \infty }||z\left(t\right)-\Phi \hat{z}\left(t\right){\left|\right|}^{2}=0, \end{array}$$

*where $\varphi_{l}\in C$ (l=1,2,…,n) is a constant, and ||·|| represents the 2-norm.*

**Remark** **2.** *The proposed CMPS contains several kinds of synchronization found in previous papers. For example, CPS will appear when $\varphi_{1}=\varphi_{2}=\cdots =\varphi_{n}=\varphi \in C$; CCS will be realized when $\varphi_{1}=\varphi_{2}=\cdots =\varphi_{n}=j$; CAS will be realized when $\varphi_{1}=\varphi_{2}=\cdots =\varphi_{n}=-j$; MPS will appear when $\varphi_{1},\varphi_{2},\cdots ,\varphi_{n}\in R$; and PS will appear when $\varphi_{1}=\varphi_{2}=\cdots =\varphi_{n}=\varphi \in R$. Consequently, CMPS is as the extension of CPS, CCS, CAS, MPS, PS, CS, AS, etc.*

Next, we investigate the process of CMPS based on a nonlinear state observer. From the error Equation (6), we obtain that

$$\begin{array}{c} D_{*}^{q}e=D_{*}^{q}z-\Phi D_{*}^{q}\hat{z}. \end{array}$$

Thus, taking into account the system (2) and the observer (4), we have

$$\begin{array}{c} D_{*}^{q}e=\Theta e+\Psi \left(f\left(z\right)-f\left(\hat{z}\right)\right)-\Psi \left(y\left(t\right)-\hat{y}\left(t\right)\right). \end{array}$$

Substituting (5) into the above equation, one can conclude that

$$\begin{array}{c} D_{*}^{q}e=\left(\Theta -\Psi K\right)e. \end{array}$$

Separating real and imaginary parts, we have two real systems as follows:

(7) $$\begin{array}{c} D_{*}^{q}e^{r}=\left(\Theta -\Psi K\right)e^{r}, \end{array}$$

and

(8) $$\begin{array}{c} D_{*}^{q}e^{i}=\left(\Theta -\Psi K\right)e^{i}, \end{array}$$

where Θ−ΨK is the linear time invariant matrix. For the sake of making systems (7) and (8) controllable, we choose the appropriate gain matrix *K* to satisfy $|arg(\lambda_{l}\left(\Theta -\Psi K\right))|>q\pi /2,$ (l=1,2,…,n). Thus, on the basis of Lemma 1, we can obtain $e^{r}\to 0$ and $e^{i}\to 0$ as t→∞, that is, $e=e^{r}+je^{i}\to 0$ as t→∞. Hence, system (2) and the observer (4) can realize CMPS.

**Remark** **3.** *The eigenvalues of matrix Θ−ΨK are independent of the complex scaling matrix *Φ*, so the complex scaling matrix *Φ* does not affect the controllability of the error systems* (7) *and* (8). *Therefore, the proposed method can arbitrarily adjust the complex scaling matrix in the synchronization process without worrying about the robustness of other synchronization methods.*

**Remark** **4.** *In this synchronization scheme, we apply the pole placement method to determine the feedback gain matrix K satisfying $|\arg(\lambda_{l}\left(\Theta -\Psi K\right))|>q\pi /2$ (l=1,2,…,n).*

**Remark** **5.** *In this paper, we only study the CMPS of FOCCS theoretically but do not study hardware implementation. Recently, there are many papers considering the implementation of the fractional-order operator and fractional-order synchronization scheme [40,41,42,43], which provide good research ideas for the implementation of the CMPS proposed in this paper. Therefore, we will further investigate the hardware implementation of the CMPS of FOCCS in future work.*

## 4. Numerical Simulations

Next, we respectively study CMPS of the following two pairs of examples to show our proposed theory.

### 4.1. CMPS of the Fractional-Order Complex Lü Systems

**Figure 1:**
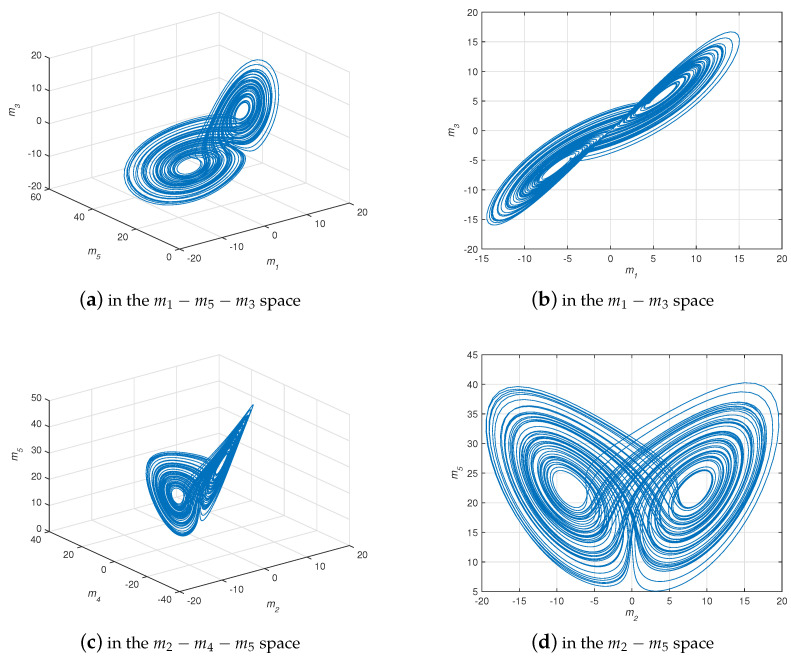
The projection of chaotic attractor for system (9) with q=0.96, β=42, γ=22, and δ=5.

The following fractional-order complex Lü system is considered as the master system, which is denoted as

(9) $$\begin{array}{c} \begin{array}{c} \left\{\begin{array}{c} D_{*}^{q}z_{1}=\beta \left(z_{2}-z_{1}\right),\\ D_{*}^{q}z_{2}=\gamma z_{2}-z_{1}z_{3},\\ D_{*}^{q}z_{3}=\frac{1}{2}\left({\bar{z}}_{1}z_{2}+z_{1}{\bar{z}}_{2}\right)-\delta z_{3}, \end{array}\right. \end{array} \end{array}$$

where β,γ,δ are real constants, $z_{1}=m_{1}+jm_{2}$, $z_{2}=m_{3}+jm_{4}$, and $z_{3}=m_{5}$ are state variables. In [6], authors found that system (9) behaves chaotically when q=0.96, β=42, γ=22, and δ=5 (see Figure 1).

Comparing system (9) with system (2), we easily have

$$\begin{array}{c} \Theta =\left(\begin{array}{ccc} -\beta & \beta & 0\\ 0 & \gamma & 0\\ 0 & 0 & -\delta \end{array}\right),\;\;\Psi =\left(\begin{array}{cc} 0 & 0\\ 1 & 0\\ 0 & 1 \end{array}\right),\;\;\Omega =\left(\begin{array}{c} 0\\ 0\\ 0 \end{array}\right),\;\;f\left(z\right)=\left(\begin{array}{c} -z_{1}z_{3}\\ \frac{1}{2}\left({\bar{z}}_{1}z_{2}+z_{1}{\bar{z}}_{2}\right) \end{array}\right). \end{array}$$

Suppose that an invertible complex scaling matrix $\Phi =\mathrm{diag}(\varphi_{1},\varphi_{2},\varphi_{3})$. Then the inverse matrix is computed as $\Phi^{-1}=\mathrm{diag}\left(\varphi_{1}^{-1},\varphi_{2}^{-1},\varphi_{3}^{-1}\right)$. Furthermore, the output of system (9) is assumed to be y=f(z)+Kz, where $K\in R^{2\times 3}$ is a gain matrix. Thus, design the following state observer of system (9):

(10) $$\begin{array}{c} \left(\begin{array}{c} D_{*}^{q}{\hat{z}}_{1}\\ D_{*}^{q}{\hat{z}}_{2}\\ D_{*}^{q}{\hat{z}}_{3} \end{array}\right)=\left(\begin{array}{ccc} -\beta & \beta \varphi_{1}^{-1}\varphi_{2} & 0\\ 0 & \gamma & 0\\ 0 & 0 & -\delta \end{array}\right)\left(\begin{array}{c} {\hat{z}}_{1}\\ {\hat{z}}_{2}\\ {\hat{z}}_{3} \end{array}\right)+\left(\begin{array}{ccc} \varphi_{1}^{-1} & 0 & 0\\ 0 & \varphi_{2}^{-1} & 0\\ 0 & 0 & \varphi_{3}^{-1} \end{array}\right)\left(\begin{array}{cc} 0 & 0\\ 1 & 0\\ 0 & 1 \end{array}\right)\left(\begin{array}{c} -{\hat{z}}_{1}{\hat{z}}_{3}\\ \frac{1}{2}\left({\bar{\hat{z}}}_{1}{\hat{z}}_{2}+{\hat{z}}_{1}{\bar{\hat{z}}}_{2}\right) \end{array}\right)\\ +\left(\begin{array}{ccc} \varphi_{1}^{-1} & 0 & 0\\ 0 & \varphi_{2}^{-1} & 0\\ 0 & 0 & \varphi_{3}^{-1} \end{array}\right)\left(\begin{array}{cc} 0 & 0\\ 1 & 0\\ 0 & 1 \end{array}\right)\left(y\left(t\right)-\hat{y}\left(t\right)\right), \end{array}$$

where ${\hat{z}}_{1}=s_{1}+js_{2}$, ${\hat{z}}_{2}=s_{3}+js_{4}$, and ${\hat{z}}_{3}=s_{5}$ are state variables.

The CMPS error is expressed as $e\left(t\right)=z\left(t\right)-\Phi \hat{z}\left(t\right)$. By means of system (9) and the observer (10), we have the following error dynamical system:

$$\begin{array}{c} D_{*}^{q}e=\left(\Theta -\Psi K\right)e=\left(\left(\begin{array}{ccc} -\beta & \beta & 0\\ 0 & \gamma & 0\\ 0 & 0 & -\delta \end{array}\right)-\left(\begin{array}{cc} 0 & 0\\ 1 & 0\\ 0 & 1 \end{array}\right)K\right)e, \end{array}$$

namely,

$$\begin{array}{c} D_{*}^{q}e^{r}=\left(\left(\begin{array}{ccc} -\beta & \beta & 0\\ 0 & \gamma & 0\\ 0 & 0 & -\delta \end{array}\right)-\left(\begin{array}{cc} 0 & 0\\ 1 & 0\\ 0 & 1 \end{array}\right)K\right)e^{r}\;\mathrm{and}\;D_{*}^{q}e^{i}=\left(\left(\begin{array}{ccc} -\beta & \beta & 0\\ 0 & \gamma & 0\\ 0 & 0 & -\delta \end{array}\right)-\left(\begin{array}{cc} 0 & 0\\ 1 & 0\\ 0 & 1 \end{array}\right)K\right)e^{i}. \end{array}$$

Assume that the eigenvalues of matrix Θ−ΨK are assigned as (−42,−2,−10), satisfying $|\arg(\lambda_{l}\left(\Theta -\Psi K\right))|>q\pi /2,\left(l=1,2,3\right)$. Based on the pole placement method, the feedback matrix *K* can be computed as

$$K=\left(\begin{array}{ccc} 0 & 24 & 0\\ 0 & 0 & 5 \end{array}\right).$$

**Figure 2:**
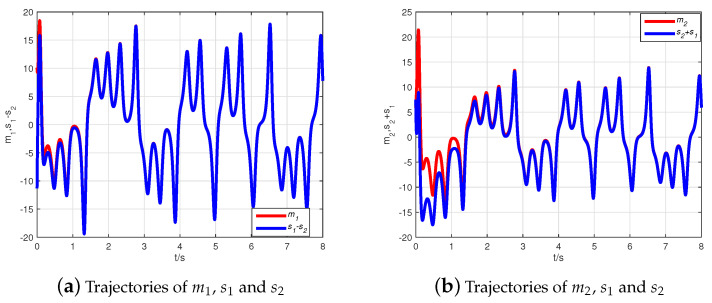

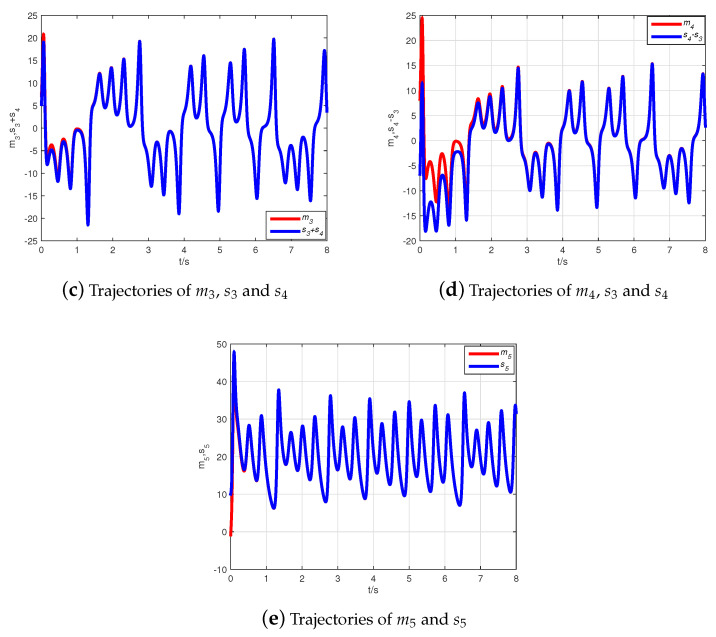
The state evolution of the system (9) and the observer (10).

**Figure 3:**
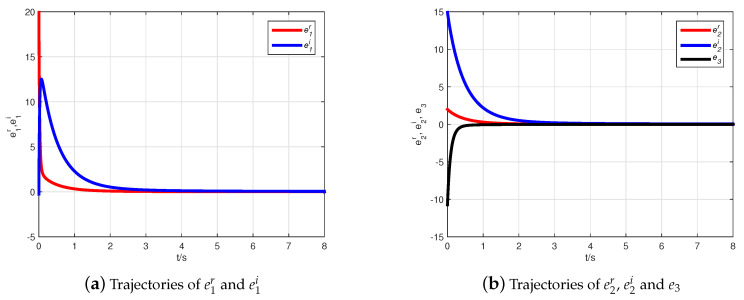
The state trajectories of the error system.

Thus, the Adams–Bashforth–Moulton predictor–corrector scheme [44] is used to obtain simulation results illustrated with the initial condition $z\left(0\right)={\left(10+6j,7+8j,-1\right)}^{T}$, $\hat{z}\left(0\right)={\left(-2+10j,6-j,10\right)}^{T}$. Choosing Φ=diag(1+j,1−j,1), the inverse matrix is computed as $\Phi^{-1}=\mathrm{diag}\left(\left(1-j\right)/2,\left(1+j\right)/2,1\right)$. Figure 2 describes the state evolution of system (9) and the observer (10). The state trajectories of the error system are demonstrated in Figure 3, where it can be seen that the error system tends asymptotically to zero very quickly. Hence, the CMPS of fractional-order complex Lü systems based on a nonlinear state observer can be realized.

### 4.2. CMPS of the Fractional-Order Hyper-Chaotic Complex Lü System

**Figure 4:**
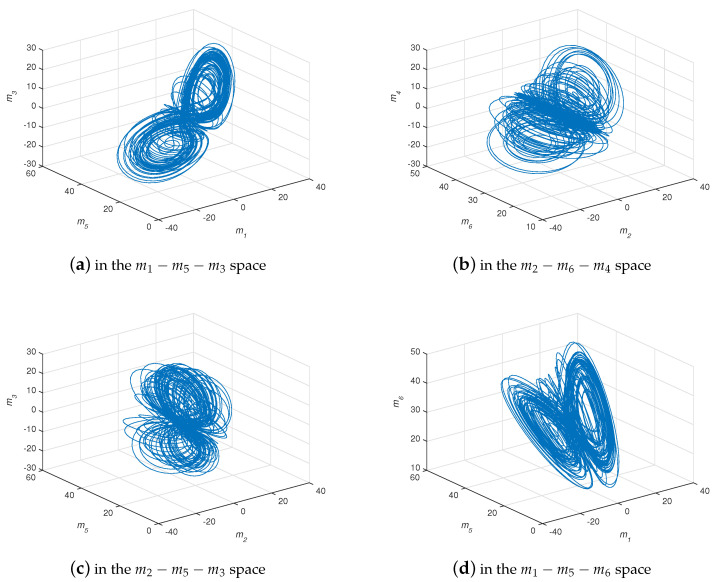
The projection of chaotic attractor for system (11) with q=0.95, β=42, γ=25, δ=6, and σ=5.

Yang and Jiang [7] firstly constructed the fractional-order hyper-chaotic complex Lü system, which reads:

(11) $$\begin{array}{c} \begin{array}{c} \left\{\begin{array}{c} D_{*}^{q}z_{1}=\beta \left(z_{2}-z_{1}\right)+z_{4},\\ D_{*}^{q}z_{2}=\gamma z_{2}-z_{1}z_{3}+z_{4},\\ D_{*}^{q}z_{3}=\frac{1}{2}\left({\bar{z}}_{1}z_{2}+z_{1}{\bar{z}}_{2}\right)-\delta z_{3},\\ D_{*}^{q}z_{4}=\frac{1}{2}\left({\bar{z}}_{1}z_{2}+z_{1}{\bar{z}}_{2}\right)-\sigma z_{4}, \end{array}\right. \end{array} \end{array}$$

where β,γ,δ,σ are real constants, and $z_{1}=m_{1}+jm_{2}$, $z_{2}=m_{3}+jm_{4}$, $z_{3}=m_{5}$, and $z_{4}=m_{6}$ are state variables. When q=0.95, β=42, γ=25, δ=6, and σ=5, system (11) generates chaotic behavior (see Figure 4).

Comparing system (11) with system (2), we easily obtain

$$\begin{array}{c} \Theta =\left(\begin{array}{cccc} -\beta & \beta & 0 & 1\\ 0 & \gamma & 0 & 1\\ 0 & 0 & -\delta & 0\\ 0 & 0 & 0 & -\sigma \end{array}\right),\;\;\Psi =\left(\begin{array}{ccc} 0 & 0 & 0\\ 1 & 0 & 0\\ 0 & 1 & 0\\ 0 & 0 & 1 \end{array}\right),\;\;\Omega =\left(\begin{array}{c} 0\\ 0\\ 0\\ 0 \end{array}\right),\;\;f\left(x\right)=\left(\begin{array}{c} -z_{1}z_{3}\\ \frac{1}{2}\left({\bar{z}}_{1}z_{2}+z_{1}{\bar{z}}_{2}\right)\\ \frac{1}{2}\left({\bar{z}}_{1}z_{2}+z_{1}{\bar{z}}_{2}\right) \end{array}\right). \end{array}$$

Suppose that an invertible complex scaling matrix $\Phi =\mathrm{diag}(\varphi_{1},\varphi_{2},\varphi_{3},\varphi_{4})$. Then the inverse matrix is computed as $\Phi^{-1}=\mathrm{diag}\left(\varphi_{1}^{-1},\varphi_{2}^{-1},\varphi_{3}^{-1},\varphi_{4}^{-1}\right)$. Furthermore, the output of system (11) is assumed to be y=f(z)+Kz, where $K\in R^{3\times 4}$ is a gain matrix. Thus, design the state observer of system (11) in the following form

(12) $$\begin{array}{c} \left(\begin{array}{c} D_{*}^{q}{\hat{z}}_{1}\\ D_{*}^{q}{\hat{z}}_{2}\\ D_{*}^{q}{\hat{z}}_{3}\\ D_{*}^{q}{\hat{z}}_{4} \end{array}\right)=\left(\begin{array}{cccc} -\beta & \beta \varphi_{1}^{-1}\varphi_{2} & 0 & \varphi_{1}^{-1}\varphi_{4}\\ 0 & \gamma & 0 & \varphi_{2}^{-1}\varphi_{4}\\ 0 & 0 & -\delta & 0\\ 0 & 0 & 0 & -\sigma \end{array}\right)\left(\begin{array}{c} {\hat{z}}_{1}\\ {\hat{z}}_{2}\\ {\hat{z}}_{3}\\ {\hat{z}}_{4} \end{array}\right)+\left(\begin{array}{cccc} \varphi_{1}^{-1} & 0 & 0 & 0\\ 0 & \varphi_{2}^{-1} & 0 & 0\\ 0 & 0 & \varphi_{3}^{-1} & 0\\ 0 & 0 & 0 & \varphi_{4}^{-1} \end{array}\right)\left(\begin{array}{ccc} 0 & 0 & 0\\ 1 & 0 & 0\\ 0 & 1 & 0\\ 0 & 0 & 1 \end{array}\right)\left(\begin{array}{c} -{\hat{x}}_{1}{\hat{z}}_{3}\\ \frac{1}{2}\left({\bar{\hat{z}}}_{1}{\hat{z}}_{2}+{\hat{z}}_{1}{\bar{\hat{z}}}_{2}\right)\\ \frac{1}{2}\left({\bar{\hat{z}}}_{1}{\hat{z}}_{2}+{\hat{z}}_{1}{\bar{\hat{z}}}_{2}\right) \end{array}\right)\\ +\left(\begin{array}{cccc} \varphi_{1}^{-1} & 0 & 0 & 0\\ 0 & \varphi_{2}^{-1} & 0 & 0\\ 0 & 0 & \varphi_{3}^{-1} & 0\\ 0 & 0 & 0 & \varphi_{4}^{-1} \end{array}\right)\left(\begin{array}{ccc} 0 & 0 & 0\\ 1 & 0 & 0\\ 0 & 1 & 0\\ 0 & 0 & 1 \end{array}\right)\left(y\left(t\right)-\hat{y}\left(t\right)\right), \end{array}$$

where ${\hat{z}}_{1}=s_{1}+js_{2}$ and ${\hat{z}}_{2}=s_{3}+js_{4}$ are complex variables, and ${\hat{z}}_{3}=s_{5}$ and ${\hat{z}}_{4}=s_{6}$ are real variables.

The error is expressed as $e\left(t\right)=z\left(t\right)-\Phi \hat{z}\left(t\right)$. By means of system (11) and the observer (12), we have the following error system

$$\begin{array}{c} D_{*}^{q}e=\left(\Theta -\Psi K\right)e=\left(\left(\begin{array}{cccc} -\beta & \beta & 0 & 1\\ 0 & \gamma & 0 & 1\\ 0 & 0 & -\delta & 0\\ 0 & 0 & 0 & -\sigma \end{array}\right)-\left(\begin{array}{ccc} 0 & 0 & 0\\ 1 & 0 & 0\\ 0 & 1 & 0\\ 0 & 0 & 1 \end{array}\right)K\right)e, \end{array}$$

namely,

(13) $$\begin{array}{c} D_{*}^{q}e^{r}=\left(\left(\begin{array}{cccc} -\beta & \beta & 0 & 1\\ 0 & \gamma & 0 & 1\\ 0 & 0 & -\delta & 0\\ 0 & 0 & 0 & -\sigma \end{array}\right)-\left(\begin{array}{ccc} 0 & 0 & 0\\ 1 & 0 & 0\\ 0 & 1 & 0\\ 0 & 0 & 1 \end{array}\right)K\right)e^{r}\;\mathrm{and}\;D_{*}^{q}e^{i}=\left(\left(\begin{array}{cccc} -\beta & \beta & 0 & 1\\ 0 & \gamma & 0 & 1\\ 0 & 0 & -\delta & 0\\ 0 & 0 & 0 & -\sigma \end{array}\right)-\left(\begin{array}{ccc} 0 & 0 & 0\\ 1 & 0 & 0\\ 0 & 1 & 0\\ 0 & 0 & 1 \end{array}\right)K\right)e^{i}. \end{array}$$

Assume that the eigenvalues of matrix Θ−ΨK are assigned as (−42,−3,−8,−6), satisfying $|\arg(\lambda_{l}\left(\Theta -\Psi K\right))|>q\pi /2,\left(l=1,2,3\right)$. Thus, based on the pole placement method, we can calculate the gain matrix *K* as follows:

$$K=\left(\begin{array}{cccc} 0 & 28 & 0 & 0\\ 0 & 0 & 2 & 0\\ 0 & 0 & 5 & 1 \end{array}\right).$$

**Figure 5:**
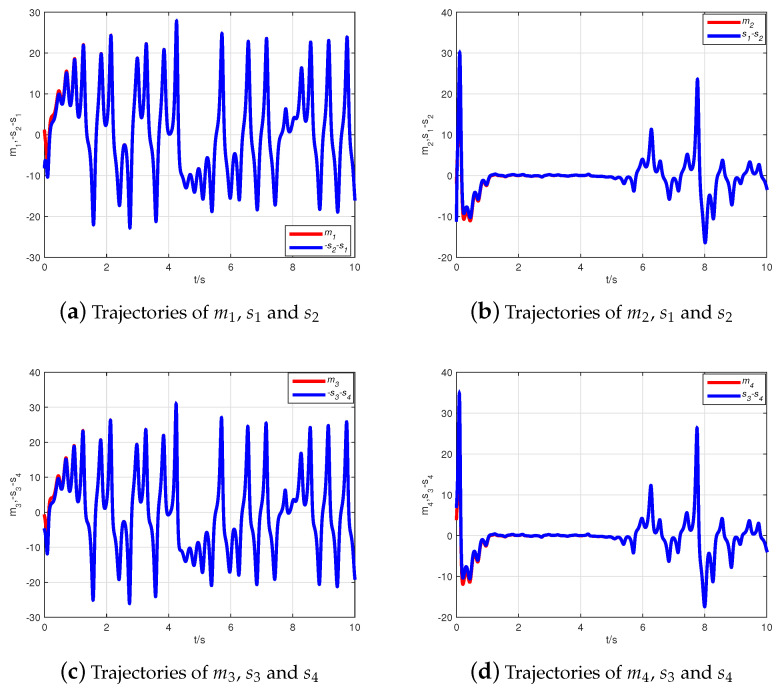

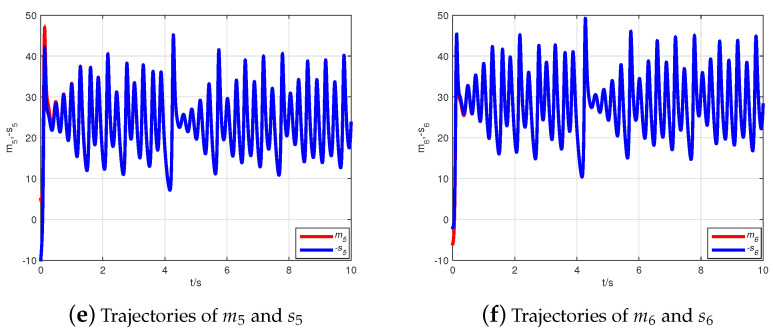
The state evolution of system (11) and the observer (12).

**Figure 6:**
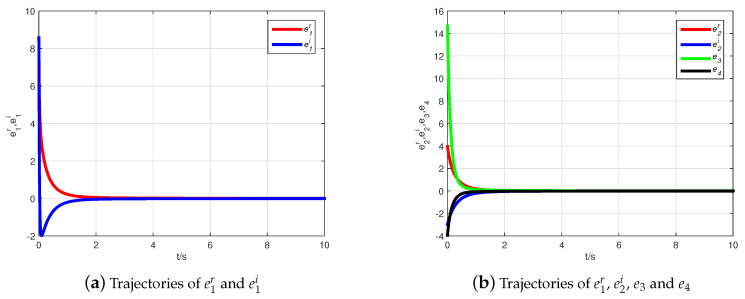
The state trajectories of the error system.

Simulation results are obtained by selecting the fractional derivative as q=0.95, the initial condition as $z\left(0\right)={\left(1-2j,-1+4j,5,-6\right)}^{T}$, and $\hat{z}\left(0\right)={\left(-2+10j,6-j,10,2\right)}^{T}$. Choosing Φ=diag(−1+j,−1+j,−1,−1), the inverse matrix is computed as $\Phi^{-1}=\mathrm{diag}\left(\left(-1-j\right)/2,\left(-1-j\right)/2,-1,-1\right)$. Figure 5 shows the state evolution of system (11) and the observer (12). From Figure 6, it is easy to see that the error system tends asymptotically to zero very quickly. Therefore, CMPS of fractional-order hyper-chaos complex Lü systems based on a nonlinear state observer can be realized.

## 5. Conclusions

This article studies the observer-based CMPS of FOCCS in detail. On the basis of the assumed output, the authors construct nonlinear state observers to realize CMPS of a large class of FOCCS. In this new synchronization scheme, it is not necessary to calculate the conditional Lyapunov exponents, and it is so effective that it can be applied in engineering. Additionally, the proposed CMPS scheme is suitable for all FOCCS, including fractional-order complex hyperchaotic systems. We respectively achieve CMPS of fractional-order complex chaotic systems: complex Lü systems, and hyper-chaos complex Lü systems. The corresponding simulation results show the correctness of this new synchronization strategy. Since CMPS has a wide application in many fields, we will consider the following two aspects in our future work: one is to extend the obtained results of this paper to other systems including impulsive systems and hybrid systems, and the other is to investigate the hardware implementation of CMPS.
